# Lipopolysaccharide Attenuates CD40 Ligand-Induced Regulatory B10 Cell Expansion and IL-10 Production in Mouse Splenocytes

**DOI:** 10.4236/oji.2015.51001

**Published:** 2015-02-12

**Authors:** Mei Lin, Jiang Lin, Yuhua Wang, Nathalie Bonheur, Toshihisa Kawai, Zuomin Wang, Xiaozhe Han

**Affiliations:** 1 Department of Immunology and Infectious Diseases, The Forsyth Institute, Cambridge, USA; 2 Department of Stomatology, Beijing Chao-Yang Hospital, Capital Medical University, Beijing, China; 3 Department of Stomatology, Fourth Hospital of Harbin Medical University, Harbin, China; 4 Department of Stomatology, Shanghai 9th People’s Hospital, Shanghai, China

**Keywords:** IL10, B10, TLR4, CD40L, LPS

## Abstract

Toll-like receptors (TLRs) play a key role in B cell-mediated innate and adaptive immunity. It has been shown that interleukin 10 (IL-10)-producing regulatory B cells (B10 cells) can negatively regulate cellular immune responses and inflammation in autoimmune diseases. In this study, we determined the effect of TLR4 signaling on the CD40-activated B10 cell competency. The results demonstrated that LPS and CD40L synergistically stimulated proliferation of mouse splenocytes. The percentage of B10 cells in cultured splenocytes was significantly increased after CD40L stimulation but such increase was diminished by the addition of LPS. Such effects by LPS were only observed in cells from WT but not TLR4^−/−^ mice. IL-10 mRNA expression and protein production in B10 cells from cultured splenocytes were significantly up-regulated by CD40L stimulation but were inhibited after the addition of LPS in a TLR4-dependent manner. This study suggests that LPS-induced TLR4 signaling attenuate CD40L-activated regulatory B10 cell competency.

## 1. Introduction

B cells have been suggested to contribute to the pathogenesis of autoimmune disease through antigen (Ag)-specific autoantibody production [1]. This central role in provoking inflammation in autoimmune diseases has made B cell attractive therapeutic targets through the depletion of these cells [2] [3]. In the past decade, however, data from a number of laboratories have revealed that B cells can also play regulatory roles in the amelioration of inflammation through their interaction with effecter T cells and other innate cells [4]. Recently an IL-10-competent CD1d(high)CD5(+) B cell subset termed B10 cells has been identified in both mice and humans that can play regulatory functions during immune and inflammatory responses [5] [6]. In most autoimmune disease models studied so far, this suppressive or regulatory role of B cells is mediated by the production of IL-10, which inhibits both Th1 and Th2 polarization, Ag presentation and pro-inflammatory cytokine production by myeloid cells [7] [8]. It also has potent activity in limiting DC function in secreting IL-6 and IL-12, and thereby inhibits Th17 cells [9]. B-cell-derived IL-10 is essential for the regulatory function of B cells, as B cells isolated from IL-10 knockout mice failed to mediate the protective function in various autoimmune disease models, such as collagen induced arthritis [10], experimental autoimmune encephalomyelitis [7], non-obese diabetes [11], and inflammatory bowel diseases [12].

CD40 ligation plays a crucial role in B cell activation, T cell-dependent antigen-driven isotype switching and germinal center formation [13]. It has been reported that selective targeting of B cells with agonistic anti-CD40 is an efficacious strategy for the generation of induced regulatory B cells and for the suppression of autoimmune disease [14]. Furthermore, recent studies have shown that B cells express distinct TLRs that determine their ability to respond to microbial patterns, which underlines their direct involvement in the regulatory functions of B cells during autoimmune and infectious diseases [15]. Understanding how B10 cells can be regulated by TLR signaling is of considerable clinical relevance in designing strategies to expand such populations to augment the treatments of autoimmune diseases and overly aggressive inflammatory responses. In this study, we investigated how TLR4 signaling affects CD40-activated B10 cell expansion and IL-10 production.

## 2. Materials and Methods

### 2.1. Mice Strain

Both groups of wild type (WT) and TLR deficient (TLR4^−/−^) mice (n = 10) were of C57BL/6 background and were used for these experiments. TLR4^−/−^ mice backcrossed to the C57BL/6 background were a kind gift from Dr. Toshihisa Kawai (Forsyth Institute, Cambridge, USA). All the mice used in the study (8 – 10 weeks old) were maintained in specific pathogen-free (SPF) units of the Forsyth Institute Animal Facility. The mice were kept on a 12-hour light/dark cycle. The experimental protocols were approved by the Institutional Animal Care and Use Committee of the Forsyth Institute.

### 2.2. Culture of Splenocytes

Mice were euthanized in a CO_2_ chamber and single-cell suspensions of splenocytes were obtained by dispersing spleen tissues through a 60-gauge stainless steel screen. Erythrocytes were removed by ACK lysing buffer (Lonza, MA). Isolated splenocytes were added into 96-well plates (2.0 × 10^5^/well) in 200 µl RPMI complete medium containing 10% FBS. CD40L (Thermo Scientific) and *E. coli* LPS (strain O55:B5, Sigma-Aldrich) were used as agonist. Splenocytes from WT and TLR4^−/−^ mice were divided into 4 treatment groups: control; *E. coli* LPS (10 µg/ml); CD40L (1 µg/ml); *E. coli* LPS (10 µg/ml) + CD40L (1 µg/ml). Cells were cultured at 37°C in a humidified incubator with 5% CO_2_ for 2 days and then were collected for analysis.

### 2.3. Cell Proliferation Analysis

Isolated mouse splenocytes were added into 96-well plates (2 × 10^5^/well) in 200 µl RPMI complete medium containing 10% FBS and were cultured for 2 days in the presence or absence of *E. coli* LPS (10 µg/ml) and/or CD40L (1 µg/ml). Cell proliferation was evaluated using a MTS reagent CellTiter 96 AQueous Assay (Promega Corp). After 4 hour incubation, the plate was read at OD 490 nm.

### 2.4. Flow Cytometry

At the termination of cell culture, splenocytes in the 96-well plates were washed with PBS followed by incubation with fluorescence conjugated antibodies, including FITC-conjugated anti-mouse CD19, PE-conjugated antimouse CD5 and Alexa Fluor 647-conjugated anti-mouse CD1d, using mouse regulatory B cell (B10) flow kit (Biolegend). At least 20,000 cells were counted for analysis and at least 100,000 cells were sorted from each sample for PCR and ELISA.

### 2.5. Reverse Transcription and Quantitative Real Time PCR

Total RNA was extracted from the cells using a Purelink RNA mini kit (Life Technology) following manufacturer’s instructions. Isolated mRNA (0.1 µg each) was reverse transcribed into cDNA using the SuperScriptII reverse transcription system in the presence of random primers (Invitrogen). Real-time PCR was carried out in a 20 µl reaction system using SuperScript III Platinum SYBR Green One-Step qRT-PCR Kit (Life Technology) in a Roche LightCycler 480 (Roche Diagnostics, Indianapolis, IN). Each RNA sample was loaded in duplicate into the plate with a template amount of 10 ng. Predesigned primers of GAPDH and IL-10 were from Sigma. The sequences of the primers used were: IL-10: 5’-agcactcccgtctcaaagaa-3’ and 5’-tgacgaacatctctggcttg-3’ (106 bp); GAPDH: 5’-ccccagcaaggacactgagcaa-3’ and 5’-gtgggtgcagcgaactttattgatg-3’ (162 bp). The real-time PCR conditions were: 50°C for 3 minutes, 95°C for 10 minutes, followed by 40 cycles of 95°C for 10 seconds, 58°C for 10 seconds, 72°C for 15 seconds. Results were presented as fold changes relative to GAPDH reference.

### 2.6. ELISA

Cell culture supernatant were collected and IL-10 level in the supernatant was detected using a Mouse IL-10 ELISA MAX Standard kit (Biolegend).

### 2.7. Statistical Analysis

All the quantitative data are expressed as means ± standard error. IL-10 gene expression by PCR, IL-10 production by ELISA were evaluated by the Student-Newman-Keuls (SNK) multiple comparison test following one-way analysis of variance (ANOVA). P values of <0.05 were considered statistically significant.

## 3. Results

### 3.1. LPS-Induced Proliferation of Mouse Splenocytes Was Enhanced by CD40L

**Figure 1:**
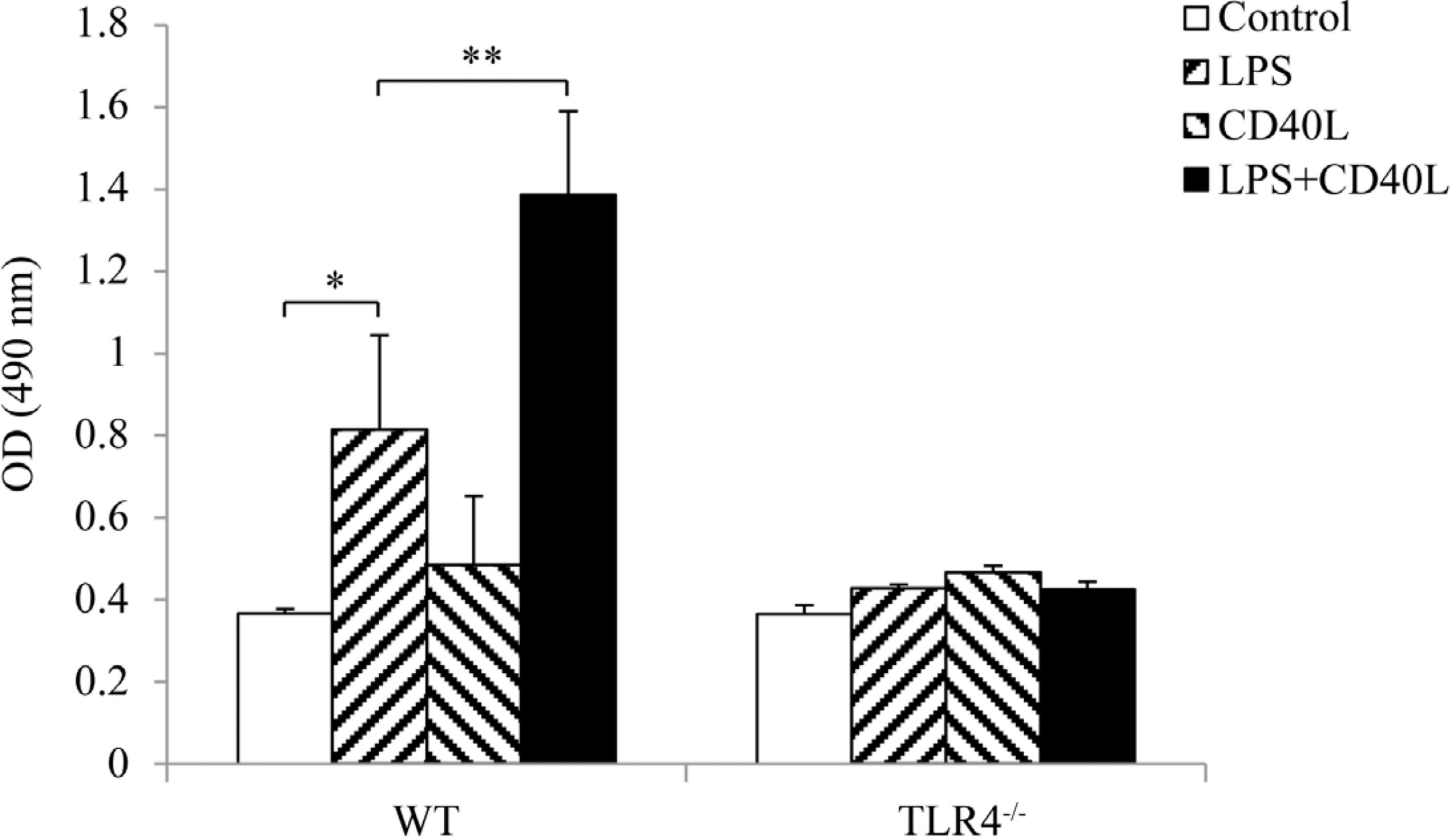
Stimulation of mouse splenocytes proliferation by LPS and/or CD40L. Cultured mouse slpenocytes were treated with *E. coli* LPS (10 µg/ml) and/or CD40L (1 µg/ml) for 2 days and the overall cell proliferation status was determined by CellTiter 96 AQueous Assay (Promega Corp). The results are representative of at least six independent experiments in duplicates. Data are presented as mean ± SEM, n = 6. Student t-test, *P < 0.05, **P < 0.01.

Cultured mouse slpenocytes were treated with *E. coli* LPS and/or CD40L and the overall cell proliferation status was determined. The results demonstrated that *E. coli* LPS significantly promoted proliferation of splenoctes from WT mice but not those from TLR4^−/−^ mice (Figure 1). Cell proliferation was not affected when cells were treated with CD40L alone, while LPS-induced cell proliferation was greatly enhanced by the addition of CD40L in WT but not TLR4^−/−^ mice (Figure 1). These results suggested that CD40L enhances LPS-induced mouse splenocytes proliferation in a TLR4-dependent manner.

### 3.2. CD40L-Induced B10 Cell Expansion in Mouse Splenocytes Was Inhibited by LPS

**Figure 2:**
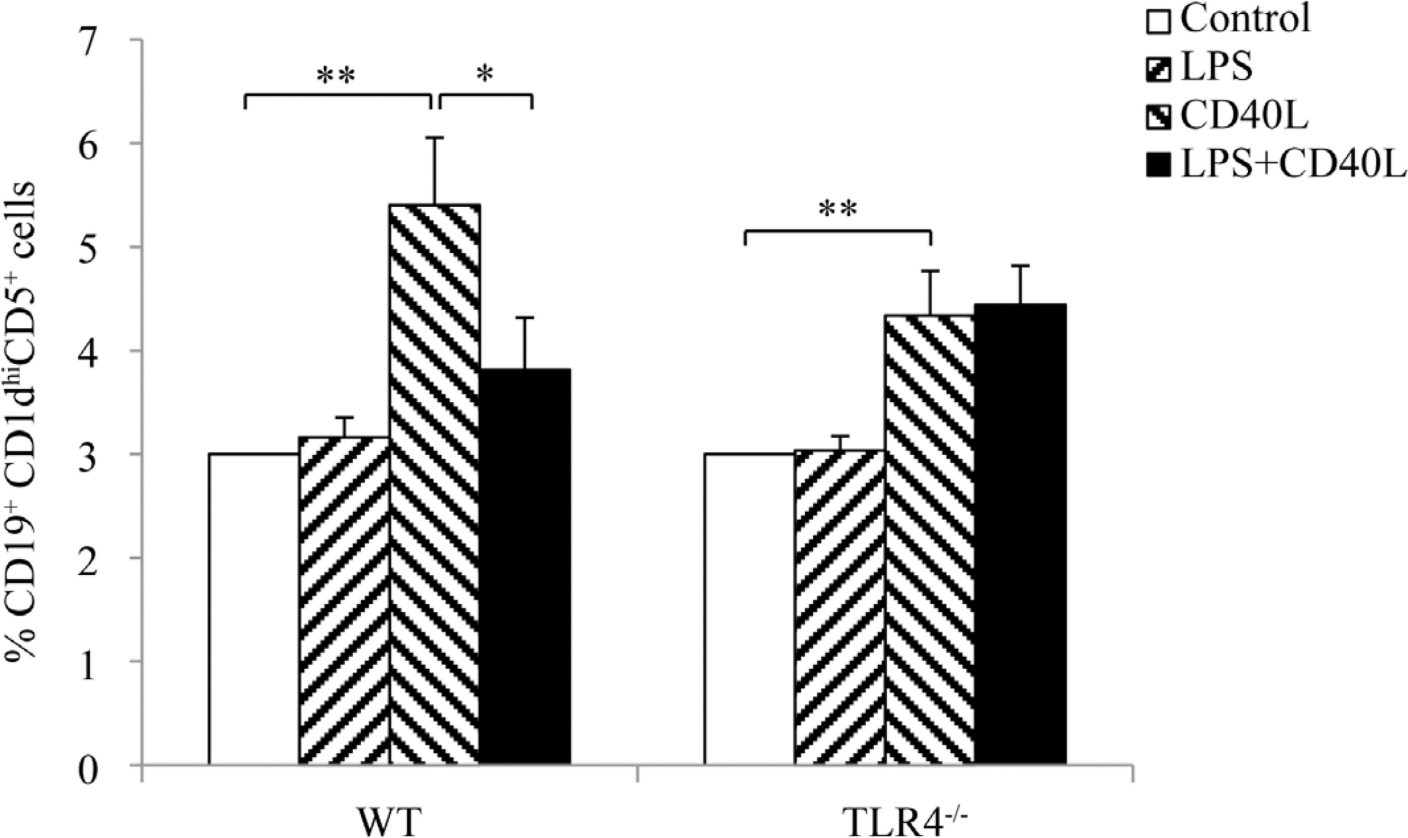
B10 cell expansion in mouse splenocytes after LPS and/or CD40L stimulation. Cultured mouse slpenocytes were treated with *E. coli* LPS (10 µg/ml) and/or CD40L (1 µg/ml) for 2 days and CD19^+^CD1d^hi^CD5^+^ cells in the cultured splenocytes were detected using flow cytometry. At least 20,000 cells were counted for analysis. Data are presented as mean ± SEM, n = 6. Student t-test, *P < 0.05, **P < 0.01.

After incubation with *E. coli* LPS and/or CD40L for 2 days, CD19^+^CD1d^hi^CD5^+^ cells in cultured splenocytes were detected by flow cytometry. The results showed that the percentage of CD19^+^CD1d^hi^CD5^+^ cells in cultured splenocytes was unchanged when cells were treated with LPS alone but was significantly increased after CD40L stimulation. Such CD40L-induced expansion of CD19^+^CD1d^hi^CD5^+^ cells was diminished when splenocytes were treated with LPS in addition to CD40L (Figure 2). However, addition of LPS did not inhibit the CD40L-induced expansion of CD19^+^CD1d^hi^CD5^+^ cells in splenocytes from TLR4^−/−^ mice (Figure 2), substantiating that such suppressive effect of LPS is TLR4-dependent.

### 3.3. CD40L-Induced IL-10 mRNA Expression by B10 Cells Was Inhibited by LPS

**Figure 3:**
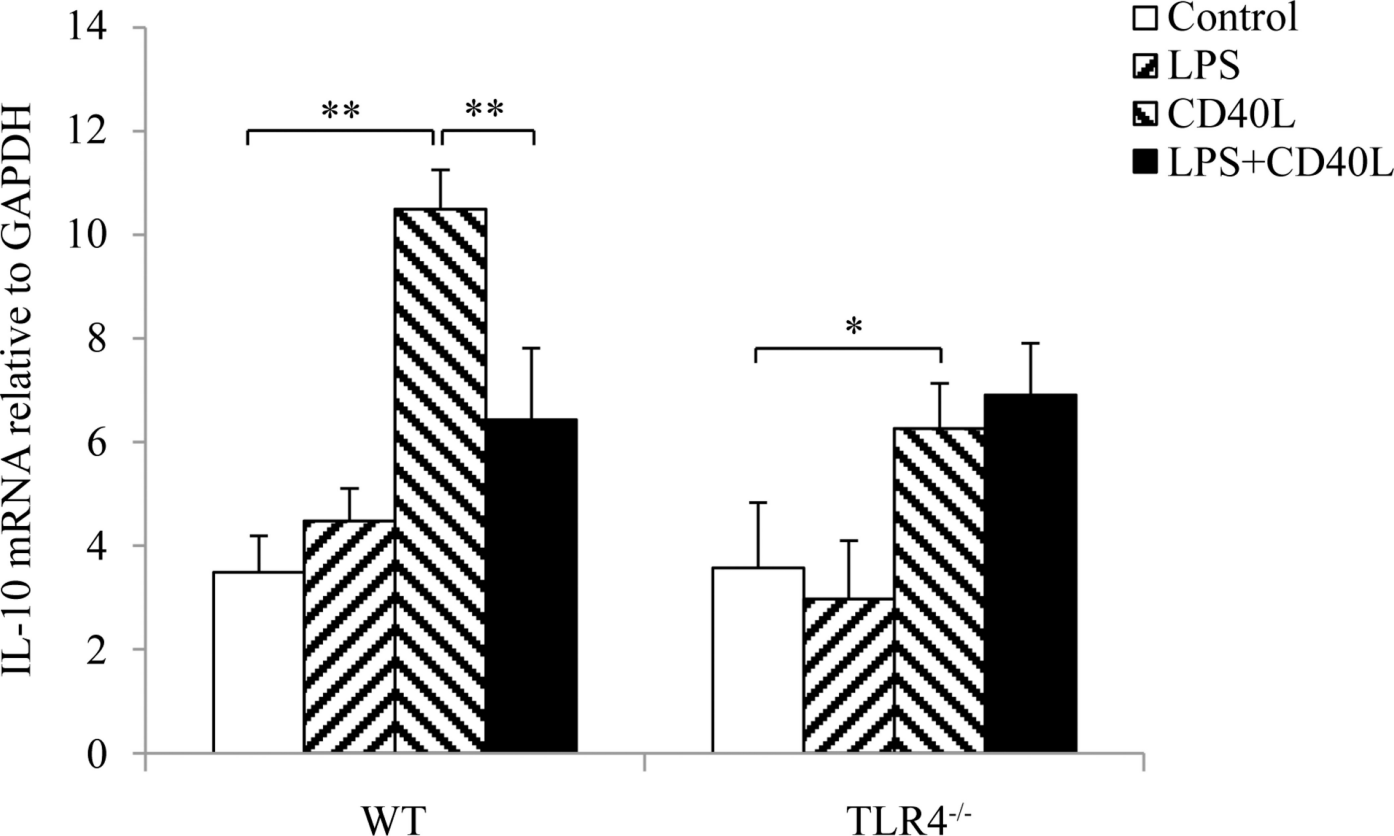
IL-10 mRNA expression by B10 cells after LPS and/or CD40L stimulation. Cultured mouse slpenocytes were treated with *E. coli* LPS (10 µg/ml) and/or CD40L (1 µg/ml) for 2 days and CD19^+^CD1d^hi^CD5^+^ cells in the cultured splenocytes were sorted out using flow cytometry. Total RNA were isolated from sorted cells using a Purelink RNA mini kit (Life Technology) and IL-10 expression was determined by real time PCR. At least 100,000 cells were sorted from each sample for PCR. Data are presented as mean ± SEM, n = 6. Student t-test, *P < 0.05, **P < 0.01.

After incubation with *E. coli* LPS and/or CD40L for 2 days, CD19^+^CD1d^hi^CD5^+^ cells representing B10 cells in cultured splenocytes were sorted by flow cytometry. After RNA isolation, IL-10 expression levels in sorted B10 cells were detected by real-time PCR. The results showed that IL-10 mRNA level in B10 cells was not affected by LPS treatment but was significantly elevated after CD40L stimulation (Figure 3). Such increase was diminished when splenocytes were treated with LPS in addition to CD40L (Figure 3). However, addition of LPS did not inhibit the CD40L-induced up-regulation of IL-10 expression in B10 cells from TLR4^−/−^ mice (Figure 3).

### 3.4. CD40L-Induced IL-10 Protein Production by B10 Cells Was Inhibited by LPS

**Figure 4:**
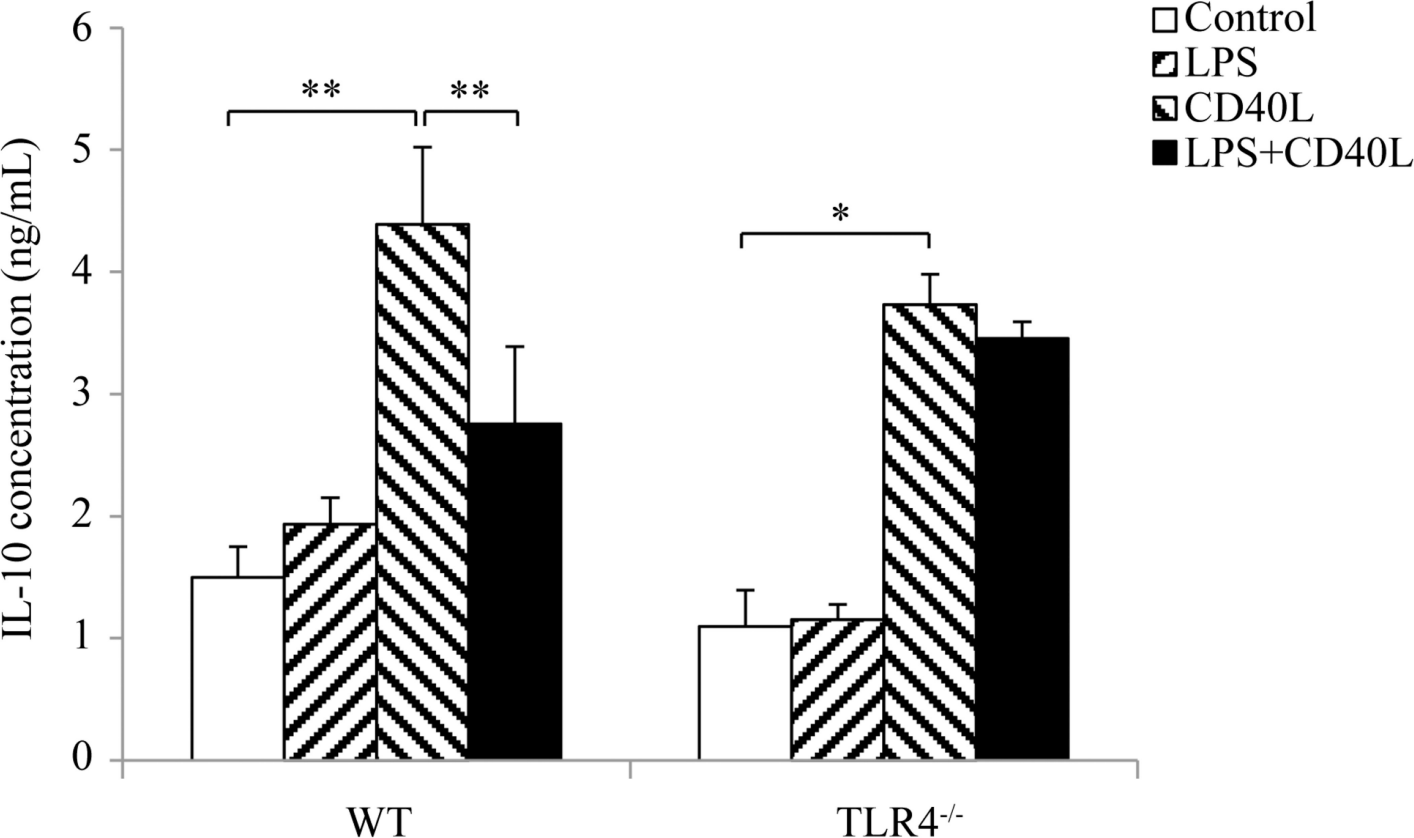
IL-10 protein production by B10 cells after LPS and/or CD40L stimulation. Cultured mouse slpenocytes were treated with *E. coli* LPS (10 µg/ml) and/or CD40L (1 µg/ml) for 2 days and CD19^+^CD1d^hi^CD5^+^ cells in the cultured splenocytes were sorted out using flow cytometry. Total protein lysate were collected from sorted cells and IL-10 production was determined by ELISA. At least 100,000 cells were sorted from each sample for ELISA. Data are presented as mean ± SEM, n = 6. Student t-test, *P < 0.05, **P < 0.01.

After incubation with *E. coli* LPS and/or CD40L for 2 days, CD19^+^CD1d^hi^CD5^+^ cells in cultured splenocytes were sorted by flow cytometry and IL-10 protein levels in sorted B10 cells were measured by ELISA. The results showed that IL-10 protein level in sorted B10 cells was not affected by LPS treatment but was significantly elevated after CD40L stimulation (Figure 4). Such increase was dramatically reduced when splenocytes were treated with both CD40L and LPS (Figure 4). However, addition of LPS did not inhibit the CD40L-induced production of IL-10 in cultured splenocytes from TLR4^−/−^ mice (Figure 4).

## 4. Discussion

TLR signaling play an important role in bridging innate and adaptive immunity mediated by B cells [16]. Although studies have suggested that agonists for TLR synergized with CD40 stimulation for T cells, little is known about the effects of the CD40 pathway together with TLR-derived stimuli in B cells. Our results suggest that TLR agonists can interact with signals from adaptive immunity to regulate B cell function during host immune response. In particular, activation of TLR4 signaling by LPS together with activation of CD40 pathway by CD40L attenuated regulatory B cell (B10) competency.

It has been demonstrated that TLR agonists synergize with CD40L to induce either proliferation or plasma cell differentiation of mouse B cells [17]. However, how TLR agonists interact with CD40L to modulate the function of regulatory B cells is completely unknown. By performing multi-parametric analysis using CD19, CD5, CD1d antibodies by flow cytometry, we were able to accurately identify low frequency and phenotypically unique regulatory B10 cells and determine their responses to the TLR agonist (LPS) and CD40L. Our results indicated that cell proliferation and regulatory B cell (B10) function are differentially regulated by LPS/CD40L. CD40L synergistically stimulates LPS-induced splenocytes proliferation while LPS antagonize CD40L-induced B10 activations, namely IL-10 production. Previous studies have indicated that TLR-mediated activation of T cells can directly promote the development of autoimmunity and TLR-4-stimulation can activate the antigen presenting cells sufficiently to deliver the signals required to drive the pathogenic function of the T cell [18] [19]. More interestingly, recent data showed that treatment with LPS selectively promoted in the recipient mice the generation of IL-6-producing activated B cells and mediated the differentiation of naive CD4 cells into Th17 phenotypes [20]. Given the pivotal role of regulatory B cells in the control of autoimmunity [7] [8], our findings may provide novel mechanism of autoimmune pathogenesis via LPS-associated suppression of CD40-activated regulatory B cells.

It is noted from our results that the level of IL-10 production detected by PCR and ELISA represent the IL-10 production by B10 cells in the context of cultured splenocytes, not purified B cells as others have demonstrated previously [6]. Yanaba *et al.* clearly showed that LPS as well as CD40 stimulation promotes B10 generation and that LPS, but not CD40, stimulation induces IL-10 secretion in purified B cells *in vitro* [6]. A possible explanation for the discrepancy between these findings and current findings could be that cellular components other than B cells, when responsive to the LPS/CD40L stimulation, are also contributed to the subsequent IL-10 production by B10 cells. B cell responses observed in this study are in the presence of other cellular components in cultured splenocytes, including T cells, dendritic cells and macrophages, which upon LPS/CD40L stimulation, provide potential co-stimulatory or counteractive molecules for the subsequent B cell activation. Therefore, the detected changes in B10 expansion and IL-10 production could be derived from both direct activation of TLR4 and CD40 on B cells and provisions of the co-stimulatory molecules by non-B cells. Studies using purified B cells directly stimulated by LPS/CD40L are warranted to verify if the observed B10 activation and IL-10 regulation in response to LPS/CD40L is solely contributed by and dependent on, intrinsic roles of B cell responses.

## 5. Conclusion

There is an emerging appreciation for the pivotal role played by B cells in several areas of human diseases including autoimmune diseases such as systemic lupus erythematosus (SLE) [21] and Sjögren’s syndrome [22]. Established B-cell-directed therapy such as Rituximab has provided a solid foundation for the assessment of the value of other B-cell-based approaches and to survey the range of B-cell involvement in human pathology [23]. The recent research advancement of regulatory B cells in human disease coincides with the vastly accelerated pace of research on the bridging of innate and adaptive immune system [24] [25]. It has been suggested that the *ex vivo* expansion of B10 cells through co-activation of innate and adaptive pathways and reinfusion of autologous B10 cells may provide a novel and effective *in vivo* treatment for severe autoimmune diseases that are resistant to current therapies [26]. Current study and our continued research may provide better understanding of the mechanisms that promote regulatory B10 cell function to counteract exaggerated immune activation in autoimmune as well as non-autoimmune conditions.

## Abbreviations

TLR: toll-like receptor
LPS: lipopolysaccharide
CD40L: CD40 ligand
IL10: interleukin 10
ELISA: enzyme-linked immunosorbent assay
